# Polysaccharide-Based Transdermal Drug Delivery

**DOI:** 10.3390/ph15050602

**Published:** 2022-05-14

**Authors:** Jingyuan Li, Hong Xiang, Qian Zhang, Xiaoqing Miao

**Affiliations:** 1 Marine College, Shandong University, Weihai 264209, China; 201900700199@mail.sdu.edu.cn (J.L.); 202017672@mail.sdu.edu.cn (H.X.); zhangqianzq@sdu.edu.cn (Q.Z.); 2 SDU-ANU Joint Science College, Shandong University, Weihai 264209, China; 3 Weihai Changqing Ocean Science Technology Co., Ltd., Weihai 264209, China

**Keywords:** polysaccharide, biocompatibility, biodegradability, penetration, diseases therapeutic, transdermal drug delivery

## Abstract

Materials derived from natural plants and animals have great potential for transdermal drug delivery. Polysaccharides are widely derived from marine, herbal, and microbial sources. Compared with synthetic polymers, polysaccharides have the advantages of non-toxicity and biodegradability, ease of modification, biocompatibility, targeting, and antibacterial properties. Currently, polysaccharide-based transdermal drug delivery vehicles, such as hydrogel, film, microneedle (MN), and tissue scaffolds are being developed. The addition of polysaccharides allows these vehicles to exhibit better-swelling properties, mechanical strength, tensile strength, etc. Due to the stratum corneum’s resistance, the transdermal drug delivery system cannot deliver drugs as efficiently as desired. The charge and hydration of polysaccharides allow them to react with the skin and promote drug penetration. In addition, polysaccharide-based nanotechnology enhances drug utilization efficiency. Various diseases are currently treated by polysaccharide-based transdermal drug delivery devices and exhibit promising futures. The most current knowledge on these excellent materials will be thoroughly discussed by reviewing polysaccharide-based transdermal drug delivery strategies.

## 1. Introduction

Transdermal drug delivery has many advantages over conventional administration, including avoiding the first-pass effect in the liver, reduced side effects, and improved patient compliance [1]. However, due to the “brick and mortar” structure of the stratum corneum, drugs cannot effectively cross the skin barrier. To surmount the cutaneous obstacle for more effective topical drug delivery, natural polymeric polysaccharides played an essential role in transdermal delivery as drug or drug delivery carriers and traditional methods such as ultrasound [2] and electrical conduction [3].

Compared with traditional carriers such as polylactic acid (PLA), poly (lactic-co-glycolic acid) (PLGA), and polyvinyl pyrrolidone (PVP), naturally derived polymeric polysaccharides performed well as drug delivery carriers with high water retention, non-toxicity, good biocompatibility, biodegradability, and many other critical biological properties. For example, chitosan (CS) has significant slow-release properties for drug delivery [4]; hyaluronic acid (HA) can increase skin hydration and involve cell signaling to promote tissue regeneration and wound healing [5]; sodium alginate could be applied for drug encapsulation by cross-linking with metal ions to prepare nanoparticles and therefore enhance skin penetration of drugs [6], etc. Further, the cross-linking or functional group modification of natural polysaccharides can change their surface properties, including hydrophilicity, hydrophobicity, mechanical strength, etc., and endow them with new functions. In addition, naturally derived polymeric polysaccharides have various pharmacological properties such as antitumor, anticoagulant, immunomodulatory, antioxidant, and anti-inflammatory. For example, *Bletilla striata* polysaccharides (BSP) have wound healing, anti-allergic, and antibacterial effects [7]; *Panax notoginseng* polysaccharides (PNPS) promote the activation of skin dendritic cells (DCs) [8]; and *Centella asiatica* polysaccharides have antibacterial and anti-inflammatory effects [9].

Polymeric polysaccharides play an influential role in treating scars, psoriasis, acne, and other skin diseases. There is an excellent potential for naturally derived polymeric polysaccharides in transdermal delivery, but a systematic summary is lacking. In this case, this review specifically presents the application of polysaccharides based on natural sources (plant, marine, microbial) as drug or drug delivery vehicles for transdermal delivery, including hydrogel, film, microneedle (MN), tissue scaffolds, and polysaccharide-based nanoparticles are developed for their targeting and good penetration ability. Diseases that require therapeutic measures, such as psoriasis, skin cancer, hypertrophic scars (HSs), etc., treated by polysaccharide-based transdermal drug delivery, are also discussed.

## 2. Polysaccharide

Polysaccharides have received increasing attention as an essential natural bioactive substance with the advantages of safety, stability, and biodegradability. They have demonstrated their unique gifts in antiviral, immunomodulatory, antioxidant, and other aspects [10,11]. In addition, the multifunctional group properties of polysaccharides make them susceptible to being modified and demonstrate more applications. Polysaccharides are broadly present in animals, plants, microorganisms, and algae. The following section will focus on several commonly used polysaccharides.

### 2.1. Herbal Polysaccharide

Herbal polysaccharides are explored to be medicines to treat various diseases due to their excellent performance in treating diabetes, hypertension, malaria, and other diseases [12,13,14]. However, traditional herbal medicines are mainly applied externally and administrated orally, limiting their therapeutic effects for these skin diseases. In this case, today, herbal medicines are investigated for transdermal drug delivery systems. Polysaccharides extracted from herbs are of increasing interest due to their extensive range of pharmacological applications, including acting on antitumor, immunomodulatory, antioxidant, and anti-inflammatory [15,16,17,18].

#### 2.1.1. Bletilla Striata Polysaccharide

BSP is the main active ingredient of *Bletilla striata*, with a relative molecular mass size of 1.35 × 105 [19]. BSP was used as a cosmetic additive to treat cracked skin and promote skin recovery [20], which had the functions of promoting wound healing [21], anti-aging [22], and antibacterial qualities [23]. BSP was used to treat chapped skin and ulcerative carbuncle acknowledged by the human body and comparatively painless to satisfy the diverse requirements in pharmacology.

#### 2.1.2. Panax Notoginseng Polysaccharide

PNPS is mainly found in saponin, the main active ingredient of *Panax notoginseng*. PNPS is obtained by grinding the plant and extracting it with ethanol, and a large amount of PNPS remains in the residue, accounting for about 3–5% of the extract. It is of great interest for various biological properties such as immunomodulatory, antitumor, antioxidant, anti-aging, and neuroprotective effects and has been used in the treatment of diseases [24,25].

#### 2.1.3. *G. lucidum* Polysaccharides

*Ganoderma lucidum*, known as “*Lingzhi*”, is a renowned traditional Chinese herb with a history of more than 2000 years. *G. lucidum* polysaccharides (GLPs) are highly abundant in *G. lucidum* cells and are used for their anti-inflammatory [26], immunomodulatory [27], and antitumor qualities [28]. In addition, GLPs have been used in combination with doxorubicin (DOX) as an additive that enhances the effectiveness of medical treatment in cancer [29].

#### 2.1.4. Others

BSP, PNPS, and GLPs, *Plantaginis Semen* polysaccharide (PSP), derived from the herb *Plantaginis Semen*, were combined with titanium dioxide nanoparticles as a new promising immune adjuvant to prevent infectious laryngotracheitis (ILT) [30]. *Radix Hedysari* polysaccharides (HPS), derived from *Radix Hedysari*, have been proved to have antitumor and antidiabetic effects [31]. *Lycium barbarum* polysaccharides (LBPs), obtained from *Lycium barbarum* L., have also been widely discussed for their anticancer effects [32]. The efficacy of more and more herbal polysaccharides is being discovered, providing good prospects for future drug therapy.

### 2.2. Marine Polysaccharide

Polysaccharides extracted from marine organisms have been extensively investigated over the last few years because they can be easily extracted from marine organisms and exhibit anti-inflammatory and antibacterial properties in drug delivery [33]. Moreover, marine polysaccharides show unique advantages in transdermal drug delivery owing to their good targeting and readiness to be modified.

#### 2.2.1. Chitosan

CS is among the most broadly employed polymers in numerous biomedical applications and has seen increased research in recent years. It is derived from chitin and is available by partial deacetylation of chitin [34]. The molecular weight of CS is between 300 and 1000 kDa. CS is the only positively charged polysaccharide in natural polysaccharides, and protonated CS can cooperate with the negative charge of the stratum corneum to improve drug penetration. The hydroxyl groups at the C-3 and C-6 positions and amino groups at the C-2 position are easily modified and facilitate various reactions [35]. The modifiability of the functional groups leads to their ability to be tailored to the desired mechanical strength and functionality. Many pH-responsive compounds based on CS are extensively applied in transdermal drug delivery [36,37]. In addition, it is considered a potential antifungal drug because of its biocompatibility, biodegradability, non-toxicity, hemostatic activity, and antibacterial and antimycotic properties [38,39,40].

#### 2.2.2. Hyaluronic Acid

HA is a linear glycosaminoglycan (GAG) consisting of *N*-acetyl-d-glucosamine and d-glucuronic acid. HA derived from rooster comb [41], microbial source [5]. HA was first isolated from the vitreous humor of bovine eyes. Studies suggest that HA may be the most widely used marine polymer in transdermal drug delivery systems [42]. HA is one of the essential components of human skin and is detected in extracellular tissues in different body parts [43,44]. HA has the properties of non-toxic, non-immunogenic, biocompatible, and high-water affinity [45]. HA is also used in various biomedical applications, such as cartilage regeneration [46], ophthalmology [47], and cancer therapy [48]. It could interact with the stratum corneum barrier to promote drug penetration. HA could also target CD44 fibroblasts, and cancer therapy with HA as a vehicle is evolving [49]. HA has been authorized by the Food and Drug Administration (FDA) as a dermal filler, showing great promise in drug delivery [50].

#### 2.2.3. Alginate

Alginate is a natural, biodegradable anionic polysaccharide, and sodium alginate is the most commonly employed one. Alginate consists of different ratios of β-Dmannuronic acid (M-blocks) and α-l-guluronic acid (G-blocks). The mannuronic blocks/guluronic acid block (M/G) ratio predominantly affects the properties of alginate [51]. Alginate with a high M/G ratio can promote chronic wound healing by producing cytokine production through human monocytes [52]. In addition, alginate also binds to metal ions through electrostatic and ionic interactions. In transdermal drug delivery systems, alginate is mainly utilized to prepare MNs [53] and nanoparticles [54]. Alginate-based MNs were used to load vaccines and deliver macromolecules such as bovine serum proteins and insulin [55,56]. Alginate was combined with CS to prepare nanoparticles possessing anti-inflammatory activity and antibacterial for the targeted therapy of cutaneous pathogens [57].

#### 2.2.4. Ulvan Polysaccharide

Ulvan polysaccharide is a biologically active natural sulfated polysaccharide with excellent properties such as antibacterial [58], antioxidant [59], antiviral [60], and hypolipidemic activity [61]. Ulvan polysaccharide mainly consists of rhamnose 3-sulfate, xylose-2-sulfate, and glucuronic acid, and is widely used as a raw material for the preparation of hydrogels [62]. Ulvan polysaccharide contains hydroxyl groups that can form hydrogen bonds, producing gel-like properties [63]. In the presence of boric acid and divalent cations, thermally reversible hydrogels can be formed [64]. Composite hydrogels prepared using ulvan polysaccharide and CS exhibited excellent cell proliferation [65].

### 2.3. Exopolysaccharide

EPSs are currently considered helpful in dealing with cancer, tumors, ulcers, etc. [66]. A fungal homopolymer, schizophyllan, produced by schizophyllum commune, has been used to treat cancer, demonstrating excellent therapeutic efficacy when present in the triple-helical form [67]. Xanthan gum (XG), produced by *Xanthomonas campestre*, is considered the most commercially available EPSs and has been prepared as hydrogels [68]. Notably, extreme environments tend to harbor specific microorganisms that secrete substances, which often have high temperature, alkali, and acid resistance in some respects. Proteins and microbial EPSs collected from these extremophiles are currently used industrially [69,70]. In this case, polysaccharides secreted by such microorganisms are to be expected.

## 3. Polysaccharide-Based Vehicles

**Figure 1:**
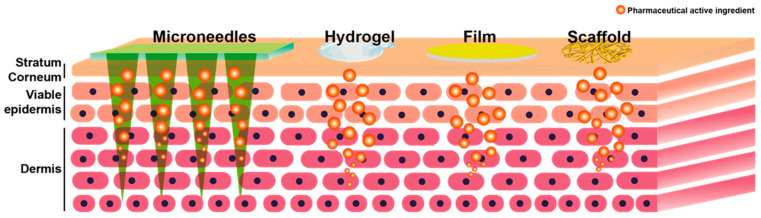
Four types of polysaccharide-based vehicles in transdermal drug delivery.

Compared with traditional polymers, polysaccharides are biodegradable and non-toxic, making them widely employed in transdermal drug delivery. Polysaccharide-based transdermal drug delivery system is also evolving, as shown in Figure 1. For example, the polysaccharide-based hydrogel shows advantages in wound dressings due to their good swelling and high hydrophilia properties; the polysaccharide-based films enable continuous drug release due to their good elasticity and breathability; the polysaccharide-based MNs have good mechanical strength and can smoothly penetrate the skin stratum corneum, and the biodegradability of polysaccharides makes MNs more biocompatible; the polysaccharide-based tissue scaffolds show unique biomimetic potential due to its good biocompatibility. The polysaccharide-based transdermal drug delivery vehicles are discussed in detail in the following section.

### 3.1. Polysaccharide-Based Hydrogels

Hydrogels are three-dimensional, hydrophilic, and polymeric networks with the ability to absorb large quantities of water. Hydrogels have received heaps of attention as outstanding candidates for bioadhesion, controlled release, and targeted therapeutic agent devices in drug delivery systems. Hydrogels prepared from conventional materials such as hydroxyethyl methacrylate are not soluble for the existence of chemical cross-linking or physical cross-linking [71]. Polysaccharide-based hydrogels are soluble and have good water retention ability and antibacterial advantages. Highly absorbent hydrogel materials prepared by cross-linking carboxymethyl agarose (CMA) and polyacrylamide (PAm) can absorb an aqueous solution hundreds of times heavier than its weight [72]. The hydrogel of BSP combined with Carbopol 940 has good viscoelasticity and physical strength and can be used in wound dressing to promote plasma coagulation and facilitate wound healing [73,74]. Polysaccharide-based hydrogels are also adopted for the delivery of macromolecules. Polysaccharide transparent hydrogel patches were used to improve the permeability of proteins to the skin. Gold nanorods are placed on the surface of the HA-based hydrogel. The temperature of the skin was raised by irradiating the gold nanorods with a laser to promote protein penetration through the skin [75].

**Figure 2:**
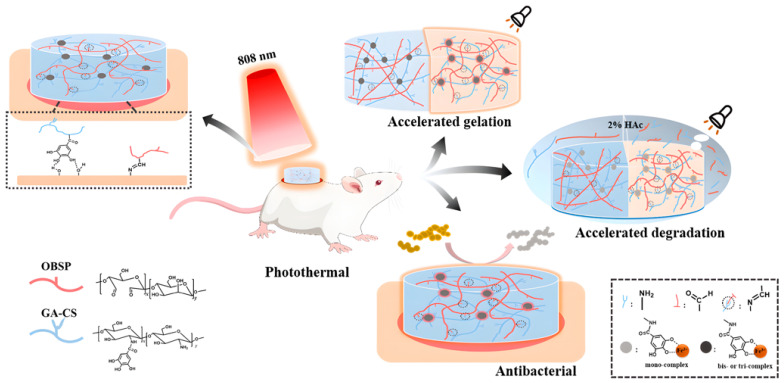
A schematic diagram of the dual network polysaccharide hydrogel and photothermal effect accelerated the gelation and degradation rate of the hydrogel and gallic acid with Fe^3+^ showed good antibacterial properties. OBSP: oxidized *Bletilla striata* polysaccharide; GA-CS: gallic acid grafted chitosan [66]. Reproduced with permission from Chonghao Chen, Carbohydrate Polymers; published by Elsevier, 2021.

Some “smart” hydrogels are continuously researched for the controlled release of drugs according to changing external conditions. Common ones are pH/temperature-sensitive hydrogels. pH-sensitive hydrogels made from hydroxyethyl cellulose (HEC)/HA complex loaded with isoliquiritigenin (ILTG) were used to treat skin disorders caused by pH imbalance [76]. Hydrogels that can be regulated by pH and temperature have also been prepared. Pluronic F-127 based pH and temperature dual-responsive hydrogels prepared with nano-conjugate of HA and CS oligosaccharide lactate were loaded with gallic acid (GA) in treating atopic dermatitis [77]. The cost-effective and versatile dual dynamic cross-linking hydrogels have been studied to facilitate wound healing and prevent infection. The hydrogels composed of oxidized *Bletilla striata* polysaccharide (OBSP), GA-grafted CS, and pyrogallol-Fe^3+^, and Schiff base influenced its cross-linking form (Figure 2). The photothermal effect and polysaccharide combination enabled the hydrogels to degrade and accelerate the gelation on demand. The cross-linking of GA with Fe^3+^ made the hydrogel have good photothermal properties. The gelation time of the hydrogel under NIR radiation was 1/2–1/3 of that without radiation. The NIR irradiation made the temperature of the hydrogel rise, and by adjusting the irradiation time and IR intensity, the antibacterial activity can be satisfied without burning the skin. The photothermal effect also accelerated the degradation of the hydrogel. NIR radiation for 20 min allowed complete decomposition of the hydrogel in 2% acetic acid solution, providing a convenient way to observe the wound. The potential antibacterial activity of GA and Fe^3+^ and the increase in the number of CS naked amine groups allowed the hydrogel to exhibit good antibacterial effects. It created a long-lasting antibacterial environment that persisted to a large extent until wound healing, showing good promise in clinical practice [78].

To summarize, hydrogels corresponding to external stimuli are constantly developing and becoming smarter. They have good prospects in transdermal drug delivery in the future.

### 3.2. Polysaccharide-Based Films

Polymeric films have attracted interest as an alternative to patches because they are transparent, flexible, non-occlusive, and easy to administer while prolonging the drug retention time in the skin [79]. The films demonstrate greater drug loading and better drug release than ointment [80]. The film-forming ability of polysaccharides and their unique adhesion properties have attracted widespread attention. In this case, the polysaccharide-based films emerge as a candidate for transdermal drug delivery vehicles.

**Figure 3:**
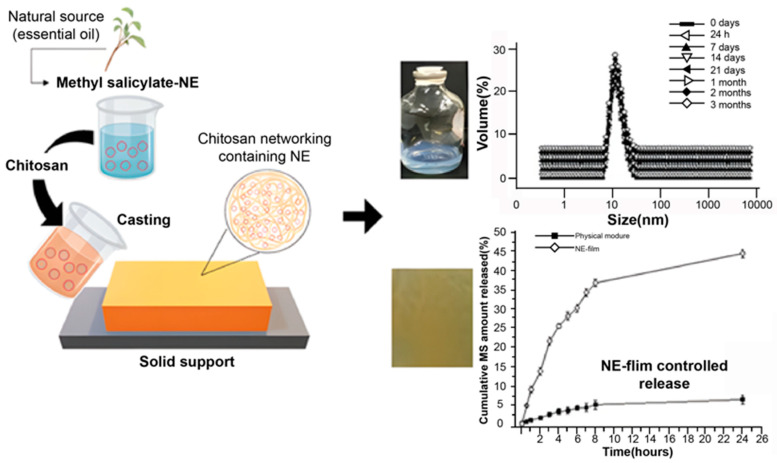
Schematic diagram of the process of nanoemulsions (NE)-film and the size and PDI analysis of formulation for three months after ultrasonication process and the release effect compared with a physical mixture for 24 h [82]. Reproduced with permission from Talita Nascimento daSilva, International Journal of Biological Macromolecules; published by Elsevier, 2020.

Films prepared with two or more polysaccharides tend to exhibit better properties. Nifedipine (NFD) polysaccharide-based transdermal films with sodium alginate and pectin as matrix polymers were developed to provide their long-term plasma concentrations [81]. CS-based films containing methyl salicylate (MS) nanoemulsions (NE) were prepared in some studies. NE-film demonstrated good MS release volume compared to the physical mixture (Figure 3). TGA and FTIR results confirmed that the encapsulation of MS into NE made the oily drugs fully integrated into the hydrophilic CS films, which increased the stability of the film [82]. Loading rifampicin into alginate and gelatin fiber-based film showed good wound healing effects [83].

New composite membrane formulations are also being investigated. Multilayer films by electrostatic interactions using alginate/CS/alginate-modified silica nanocapsules (SNCs) and CS biopolymers were prepared. They encapsulated Fulvestrant, a selective estrogen receptor downregulator, in SNC and then incorporated it into the film. The effectiveness of the film was releasing the drug influenced by the external pH. At pH 7.4, the film can entrap Fulvestrant well, while at pH 5.0, it releases the drug rapidly. The rate of drug release can be changed continuously according to the external pH change [84]. In the following study, HA and temperature-responsive micelles were used to prepare a sandwich-like membrane that utilized the protonation and deprotonation reactions of the micelle core for the controlled release of anticancer drugs osimertinib [85]. A layer-by-layer self-assembly method using sodium cellulose sulfate (NaCS), chitosan hydrochloride (CHC), and sodium tripolyphosphate (STPP) was used to prepare “sandwich structure” hydrogel film for loading ibuprofen (IBU). The results showed that the film penetrated the skin of mice with good controlled drug release [86]. Tri-layers prepared from sodium alginate and poly (4-vinyl pyridine) can be stabilized in an acidic buffer at pH 4.2 and used for skin wound healing under acidic conditions [87]. Ternary blend films of CS, polyethylene oxide (PEO), and levan prepared by the solution casting method demonstrated better biocompatibility than the CS-PEO binary films [88]. The weight ratio of different polysaccharides in films also affects the release of drugs [89].

### 3.3. Polysaccharide-Based Microneedles

MNs technology has attracted the attention of researchers as a minimally and efficient invasive way of drug delivery. Traditional MNs made from solids, such as silicon and metals, typically create microchannels on the skin surface where the drug enters, limiting the drug utilization [90]. Polysaccharides have the biodegradability advantage and are similar to the components of the extracellular matrix [91]. In this case, soluble MNs with polysaccharides as raw materials are continuously studied and used to enhance MN biocompatibility and improve patient compliance. The recent examples of polysaccharide-based MNs are listed in Table 1, including the basic components, pharmaceutical ingredients, and applications.

Some natural polysaccharides such as HA [92], carboxymethyl cellulose [93], alginate, maltose, and CS [94] are widely used to prepare soluble MNs. Calcium ion cross-linking alginate/maltose composite MNs loaded with insulin had a significant hypoglycemic effect compared with traditional transdermal injection [56]. Bacillus Calmette–Guérin polysaccharide nucleic acid MN patches (BCG-PSN MNP) were prepared by incorporating the ribonucleic acid fraction of the BCG vaccine into a sodium hyaluronate (HNA) based MN patch and were used for immunotherapeutic treatment. The BCG-PSN MNP exhibited increased IFN-c and TNF-a production in peripheral blood CD4+T cells [95].

**Figure 4:**
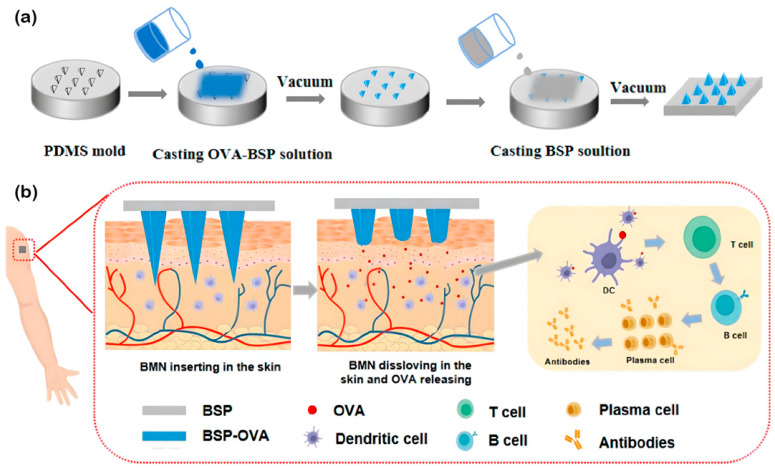
Schematic diagram of the preparation process and ovalbumin (OVA) delivery condition. (**a**) Two-step preparation of *Bletilla striata* polysaccharide (BSP) microneedle (MN) (BMN). (**b**) Transdermal drug delivery of the OVA-loaded BMN system and the release of the drug can be triggered by the dissolving of BSP in the skin interstitial fluid [97]. Reproduced with permission from Ping Zhou, International Journal of Biological Macromolecules; published by Elsevier, 2022.

Most herbal medicines have the inherent properties of promoting wound healing, and antiseptic and anti-inflammatory properties. Combined with this feature, MNs based on herbal polysaccharides have been continuously investigated in recent years. In 2018, BSP was used for the first time in the preparation of MNs [96], and BSP MN (BMN) containing Rhodamine B (RB) demonstrated good mechanical strength and a desirable cumulative penetration rate. Subsequently, BMN was used to deliver vaccines (Figure 4). The process of preparing MNs has two steps. First, the OVA/BSP solution is poured into the mold and centrifuged to form the MN tip, and then the remaining solution is removed. The BSP solution (15% *w*/*v*) was poured, centrifuged, and dried to form the BMN substrate. The BSP in the tip gave the BMN a mechanical strength of 0.63 N/needle and enabled it to penetrate the stratum corneum. The BSP in the substrate relied on its specific anti-inflammatory and antibacterial activity to promote the healing of the microchannels caused by the BMN. BMN was cytocompatible, less irritating to the skin, promoted cell growth to a certain extent, and had good low hygroscopicity. The OVA of 76.74% was released within three hours, and BMN loaded with antigen ovalbumin (OVA) maintained an intact secondary structure within 21 days [97].

**Table 1:** Some latest polysaccharide-based MNs.

Composition	Pharmaceutical Active Ingredient	Application	Main Achievement	Ref.
BSP	OVA	Infectious diseases	Better mechanical strength and stability than HA-MNs and PVA-MNs, well-reserved OVA at 4 °C for 21 days	[97]
CD-MOF, QUE, BSP	HSF membrane	Hypertrophic Scars	The combination of bio nanoparticles and soluble MNs inhibited collagens I and III expressions	[98]
BSP	RB	Drug delivery	The transdermal effect was more effective than the patch, had better mechanical strength, and promoted wound healing	[96]
PNPS	Dox, 5-Fu	Skin dendritic cell activation	It targeted skin dendritic cells, activated immune cells, and triggered T cell immune response mediated by DCs	[99]
DCS	DCS	Hemostasis	Pagoda-like shape, the insect-foot-inspired multilayer structure helped MNs adhere to the bleeding area	[100]
CS	meloxicam	Pain management for cattle	Indicated for pain control in cattle after routine surgery	[101]
CS	Insulin in a macroporous alumina core	Diabetes mellitus	The dissolution of the gel Intelligent controlled the release of insulin according to in vivo glucose level, and kept normoglycemia stable for 5 h	[102]
CS	Mg, PNS	Chronic wounds	It promoted neovascularization in chronic wounds and regulated macrophage phenotype conversion to reduce inflammation	[103]
HA, PVP	Propranolol Hydrochloride	IH	About 100% propranolol hydrochloride was released in 20 min	[104]
HA, CuS into ZIF-8	CPT	Melanoma	Achieve long-lasting enrichment at the tumor site, and the scab disappeared within 7–10 days	[105]
HA	Shikonin	HSs	HA MNs markedly reduced the proliferation and viability of HSF and downregulated fibrotic-related genes such as TGF-β1, FAP-α, and COL1A1	[106]
HA	MXD	Alopecia	HA and MXD had a synergistic effect in treating alopecia, which maximized the effectiveness of the treatment and minimized the side effects of MXD for alopecia	[107]
Alg-ABA, chondroitin sulfate	Mineralized insulin particles, GOD	Diabetes mellitus	The H^+^ produced by the reaction of GOD with glucose gradually dissolved mineralized insulin particles, leading to the self-adjustable release of insulin	[108]
Ulvan	FITC-BSA, R6G	Drug delivery	Enhance the cumulative release of FITC-BSA and biocompatibility, and it dissolved in only 2 min in porcine skin	[109]

BSP: *Bletilla striata* polysaccharide; OVA: Ovalbumin; CD-MOF: The cyclodextrin metal-organic framework; QUE: Quercetin; HSF: Hypertrophic scar fibroblast; RB: Rhodamine B; PNPS: *Panax notoginseng* polysaccharide; DOX: Doxorubicin; 5-Fu: 5-fluorouracil; DCS: Dodecyl-modified chitosan; CS: Chitosan; PNS: *Panax notoginseng* saponins; HA: Hyaluronic acid; PVP: Polyvinyl pyrrolidone; PVA: Polyvinyl alcohol; IH: Infantile hemangioma; ZIF-8: Zeolitic imidazolate framework-8; CPT: Camptothecin; HSs: Hypertrophic scars; MXD: Minoxidil; Alg-ABA: 3-amino-phenylboronic acid-modified alginate; GOD: Glucose oxidase; FITC-BSA: Bovine serum albumin–fluorescein isothiocyanate conjugate; R6G: Rhodamine 6G.

### 3.4. Polysaccharide-Based Tissue Scaffolds

Polysaccharides have been widely used for tissue scaffolds due to their good biocompatibility and biodegradability [110]. In bone tissue engineering field, different polysaccharides exhibit different excellent properties. CS can promote the attachment, proliferation, and mineralization of osteoblasts in vitro and activate endogenous bone regeneration [111,112]; cross-linking modified HA can increase the porosity to meet different strengths of bone tissue scaffolds, HA-based scaffolds show a synergistic effect with stem cells in tissue engineering [113], alginate is more suitable for cell attachment by modification of chemical bonds [114], and XG also shows a unique bionic potential in bone tissue engineering applications [115]. Nano hydroxyapatite particles (nHAP) modulate the biomineralization process of inorganic nanoparticles inside bone by functionalizing CS with a graphene oxide (GO) network matrix, which crystallizes in situ into a graphene oxide/chitosan/nHAP (GO/CS/nHAP) scaffold. The scaffolds exhibit good cell proliferation capacity and bioactivity and are considered an approach for endogenous bone repair [112].

Polysaccharides allow scaffolds to exhibit better performance and provide a good framework for drug release. Hydrogels are widely used as scaffolds for tissue engineering. Composite scaffolds prepared from alginate-based hydrogels and gelatin-based electrospun mats exhibited better mechanical strength and controlled drug release [114]. Epigallocatechin-3-gallate (EGCG) has the ability to enhance the differentiation of mesenchymal stem cells (MSCs) into osteoblasts, and pour EGCG is easily metabolized by cells and reduces bioavailability. EGCG-loaded CS nanoparticles were encapsulated into CS/alginate (CS/Alg) scaffolds (CS/Alg-ECN) to improve the utilization of EGCG. CS/Alg-ECN can activate the Wnt/β-catenin signaling pathway to promote the differentiation of osteoblasts [116]. Hydrogels made from combinations of different ratios of polysaccharides can be used as a base material for skin scaffolds and show efficient osteoinduction [117,118].

## 4. Polysaccharide-Based Penetration

The barrier effect of the stratum corneum is the key problem faced by transdermal drug delivery, and how to enhance drug penetration is the main obstacle that affects the development of transdermal drug delivery. Physical methods have been used to enhance drug penetration, but their effectiveness is limited and may lead to skin damage; in addition, physical methods often require additional equipment, resulting in poor patient compliance. Polysaccharides exhibit unique advantages in promoting drug penetration, such as charge effect, and hydration effect. Both polysaccharide permeation enhancers and polysaccharide-related nanotechnology have promoted drug penetration.

### 4.1. Penetration Enhancers

The primary problem faced by transdermal drug delivery is the stratum corneum barrier. In addition to physical penetration methods such as ultrasound, temperature, electricity, and magnetic fields [119], chemical penetration enhancers are also used to promote drug penetration into the stratum corneum, such as fatty alcohols and fatty acids [120]. Chemical penetration enhancers display some skin irritation, possibly leading to the development of inflammation and erythema [121,122]. In this case, polysaccharide-based penetration enhancers are continuously used in transdermal drug delivery systems. CS, the only positively charged polysaccharide among natural polysaccharides, is bound tightly to the negatively charged sites on the epithelial cell membrane. Its positive charge leads to the depolymerization of F-actin and the dissolution of the tight junction protein ZO-1, thereby promoting penetration [123]. HA improves the hydration of the stratum corneum, promoting penetration [124]. The high moisturizing properties of XG promote the drug’s hair follicle penetration [125]. The mucilage polysaccharide extracted from *Hibiscus rosa–Sinensis L.* forms non-covalent bonds with skin tissues, affecting drug penetration [126]. Thiolated CS opens tight junctions through interaction with the thiol groups of cysteine-containing membrane receptors [127].

### 4.2. Polysaccharide-Based Nanoparticles

Nanotechnology mainly refers to controlling the particle size of the drug at the nanoscale, which has the advantages of improving drug solubility and stability and enhancing the curative efficacy [128]. Based on the advantages of polysaccharides’ natural targeting, hydration function, and charge interaction with the skin, polysaccharide-based nanoparticles show a better prospect than ordinary nanoparticles. A diversity of methods using nanotechnologies has been investigated to optimize the efficiency of transdermal drug delivery, and several currently used methods are described below.

#### 4.2.1. Emulsion

**Figure 5:**
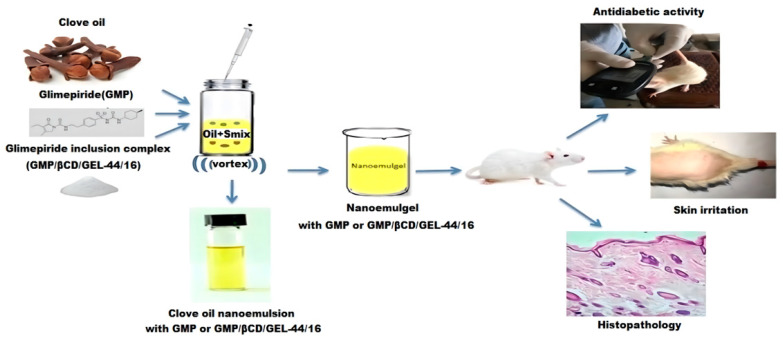
Schematic diagram of the preparation of the NE and its hypoglycemic effect, skin irritation, and in vivo antidiabetic evaluation [129]. Reproduced with permission from Fizza Abdul Razzaq, Cells; published by MDPI, 2021.

The polysaccharide-based emulsion is a thermodynamically stable colloidal system composed of the oil phase and water phase and stabilized by surfactants or cosurfactants. The pro-permeation effect of NE was used to deliver a hypoglycemic drug, Glimepiride (GMP). The NE was prepared from clove oil, Tween-80, and PEG-400, gelated with XG. It improved skin permeability and hypoglycemic activity, providing a new option for the treatment of diabetes (Figure 5) [129]. The ratio of polymer to surfactant has been proved to influence the permeation effect. Researchers prepared a microemulsion of HA and collagen and found that the molecular weight of collagen and HA did not affect the delivery efficiency [130].

Some polysaccharides with natural pharmacological activity are combined with NE to load drugs. The antifungal properties of CS were combined with the antifungal drug Fluconazole (FZ), essential oils, and sucrose fatty acid esters to prepare gel microemulsions for the treatment of mycoses [131]. CS emulsions loaded with 5-fluorouracil (5-FU) are considered a promising method for delivering 5-FU. CS facilitates the movement of 5-FU through the stratum corneum by altering the arrangement of phospholipids in the epithelial cell membrane [132]. Pickering emulsions stabilized by CS/collagen peptides nanoparticles were penetrated into the deeper stratum corneum due to the interaction of protonated CS with the negatively charged sites of the stratum corneum [133]. NE containing HNA and indomethacin (Ind) demonstrated better skin penetration and drug deposition than the HNA-Ind solution. In addition, it had an anti-inflammatory effect on ear edema in mice to a certain extent [134].

#### 4.2.2. Ethosomes

Similar to liposomes, ethosomes (ES) have a phospholipid bilayer. In addition, ES have unique properties such as high deformability and fluidity due to their relatively high concentration of ethanol (20–45%) [135,136,137], exhibiting a better effect in promoting penetration than traditional liposomes [138]. However, the promotion of permeation also leads to the problem of easy drug leakage. In this case, polysaccharides are chosen to combine with ES to enhance the stability of the formulation.

**Figure 6:**
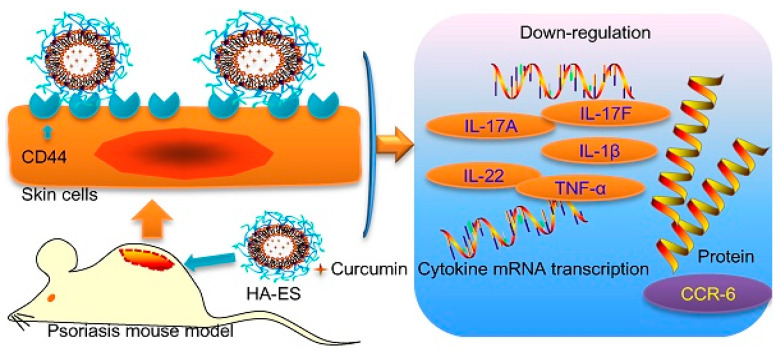
Schematic illustration of the curcumin-loaded HA-ES targeted CD44 protein and reduced the level of TNF-α, IL-17A, IL-17F mRNA, etc., and lowered the expression of CCR-6 protein [141]. Reproduced with permission from Yongtai Zhang, THERANOSTICS; published by Ivyspring International, 2019.

Polysaccharides are used to modify ES to improve their susceptibility to drug leakage. HA-modified ES (HA-ES) formed a hydrogel network on the surface of ES to reduce drug leakage, and HA-ES loaded with eugenol (EUG), and cinnamaldehyde ([EUG/CAH]) (volatile oil medicines) demonstrated better encapsulation ability and better stability compared to ES. Pharmacokinetics showed that EUG and cinnamic acid (CA) concentrations in subcutaneous tissues were considerably higher in the HA-ES group than in the ES group. In addition, the moisturizing ability of HA enhanced the hydration of the stratum corneum and facilitated transdermal drug delivery [139]. HA/ES-aminolevulinic acid (ALA) (HA/ES-ALA) with a synergistic effect was prepared by combining HA gels and ES of 5-ALA (ES-ALA). HA/ES-ALA protected ES-ALA during permeation, then HA/ES-ALA actively aggregated on the hypertrophic scar fibroblast (HSF) surface using HA receptors to release ES-ALA, and finally, ES-ALA on the surface of HSF delivered ALA into HSF through the membrane fusion mechanism [140]. HA was combined with glycol-based ES to prepare a drug carrier, HA-ES, to transport curcumin (Figure 6). The HA gel network on the surface reduced curcumin leakage, and its eight-hour cumulative transdermal volume was 1.6 times higher than that of ES. Due to the specific targeting of HA-ES on CD44 exhibited higher intradermal drug accumulation, and the levels of TNF-α, IL-17A mRNA, and CCR-6 protein were also reduced [141].

#### 4.2.3. Lipid Nanoparticles

Lipid nanoparticles (LNPs) consist of a monolayer of surfactants with a lipophilic nucleus, which is different from liposomes. At present, there are three types of LNP used for drug delivery: lipid nano-emulsions (LNE), solid lipid nanoparticles (SLN), and nano-lipid carriers (NLC). They can enhance drug permeability and have a good retention effect [142]. Bilosomes (BLS) are novel lipid nanocarriers composed mainly of amphiphilic bile salts (ABS). CS-modified bilosomes containing terbutaline sulfate (TBN) exhibited good encapsulation efficiency, with an approximately 2.33-fold increase in bioavailability compared to oral solutions [143]. However, the LNP aqueous dispersions exhibit unsuitable rheological properties. In this case, some studies combined LNP with other substances to modify this property, such as LNP-hydrogel systems. Lipophilic drugs can be efficiently loaded into the LNP, encapsulating LNP in a hydrogel network [144].

XG was added to the LNP-poloxamer hydrogel to enhance the mucoadhesive properties during the synthesis of poloxamers [145]. CS-LNPs loaded with IBU were packed into the hydrogel for the transdermal delivery of IBU. CS interacted with negatively charged IBU to form drug-polymer complexes, and in addition, the bioadhesive nature of CS improved the residence time of IBU at the application site [146]. This form of double encapsulation strategy provides better control of drug release. Although the LNP-hydrogel system shows great promise, the mechanism of drug release needs to be further investigated.

#### 4.2.4. Nanoassemblies

Due to the potential applications of nanoscale polymer in the biomedical field, many efforts have been committed to designing nanoscale polymer assemblies. The currently commonly used self-assembly method of amphiphilic copolymers usually leads to the ability of core/shell nanostructures to carry dipolar drugs in their dipolar cores [147].

Self-assembled polysaccharide-based nanoassemblies were prepared using N-alkylaminated chitosan (NACs) to deliver the anti-inflammatory drug Voltaren. The modified NACs had amphiphilic characteristics and could voluntarily assemble into nanoaggregates at particular concentrations because of the added hydrophobic aliphatic side chains of CS. The capacity of NACs to load diclofenac under a lipid environment was verified by adding almost insoluble diclofenac into paraffin oil. The solubility of diclofenac in an aqueous solution was improved by NACs [148]. Negatively charged lecithin and positively charged CS interacted electrostatically to form nanoparticles for coating drugs and achieve self-assembly at the supramolecular level forming lecithin-CS hybrid nanoparticles, thereby enhancing drug penetration to treat psoriasis. The positive charge on the surface of CS increased the deposition of nanoparticles in the skin. Compared to commercially available products, lecithin-CS hybrid nanoparticles showed faster control of psoriasis [149]. Lecithin/CS nanoparticles (LCNs) were also used to load baicalein-phospholipid complexes to form BPC-LCNs. Phospholipids combined with baicalein to form a complex and enhance the solubility of baicalein, which is encapsulated into lecithin/CS nanoparticles to enhance skin penetration [150]. Polyunsaturated fatty acids (PUFAs) and the photosensitizer chlorin e6 (Ce6) were self-assembled into nanoassemblies to prepare L-Ce6 NAs and incorporated into fast-dissolving oligo-HA MN patches to prepare L-Ce6 MNs. Combining the tumor-targeting function of HA with photodynamic therapy (PDT) allowed L-Ce6 MNs loaded with very low doses of photosensitizers to show promising results in melanoma treatment [151].

#### 4.2.5. Omniphilic Nanocarriers

The main problem with drug delivery is that it is often necessary to transfer from one phase to another, such as the transfer between the aqueous phase and lipid phase. This situation would affect the delivery of medications. Therefore, researchers were investigating if it is possible to develop a nanoparticle carrier that can deliver drugs in different phases without affecting too many properties of the drug. In this case, the concept of the omniphilic nanocarrier was proposed. These nanocarriers are named “omniphilic”, meaning “like everything”, to illustrate their ability to accommodate all kinds of molecules and adapt to solvent environments.

Nanocarriers based on the biopolymer CS encapsulate both hydrophilic and hydrophobic drugs and convey them into lipid or aqueous environments. The results exhibited that omniphilic polysaccharide-based nanocarriers (OPNs) showed excellent self-regulation ability in media with different polarities and successfully encapsulated different guest molecules in lipid or water environments, making it possible to cross the barriers between different phases. In addition, OPNs exhibited structural plasticity and adaptiveness, which allowed them to actively load drugs and achieve cross-phase transport. Based on the advantages of OPNs, for fields where nanoparticles can be used, such as the cosmetic industry, agriculture, and transdermal delivery of drugs, the combination of OPNs allows for a better performance of delivering drug molecules to the site of action and improved drug utilization [152].

## 5. Polysaccharide Based Drug Delivery for Diseases Therapeutics

Transdermal drug delivery systems allow a controlled release rate of drugs [153], and they have prospective applications in personalized medicine, matching each patient to the most appropriate medical regimen [154]. Polysaccharides are natural polymers with targeting, modifiable, inherent antibacterial, antioxidant, and other properties. These properties make them desirable compounds for biomedical applications. When drug delivery systems are modified by polysaccharides, receptors on target cells trigger phagocytosis, producing active targeting effects [155]. Polysaccharide-based transdermal drug delivery systems are now widely used in the medical field and in combination with other drugs to address therapeutic diseases, such as immunotherapy [156], diabetes [56], psoriasis [157], etc. They are described in more detail in the following sections.

### 5.1. Vaccination

Polysaccharides were used in vaccine development fifty or sixty years ago, and the capsular polysaccharide vaccine was used to prepare of anti-streptococcus pneumoniae at that time. It has become common sense to construct polysaccharide-based antimicrobial vaccines and commercialize several polysaccharide-based vaccines. For example, Ac Vax^®^, Pneumovaxll^®^, and Typhim Vi^®^ were respectively formulated against *Neiseria meningitidis, Streptococcus pneumoniae*, and *Salmonella typhi* [158,159]. In order to overcome poor immunogenicity, polysaccharides were coupled to immunogenic protein carriers and acted as part of the vaccine formulation [160]. The immunogenicity of the conjugated polysaccharide vaccine was related to the length of the polysaccharide, and the length of the Vi polysaccharide has a direct effect on the secretion of anti-Vi lgG [161]. In addition, polysaccharides with unique properties are also used in the vaccine field. For example, trimethyl chitosan, a derivative of CS, was regarded as an adjuvant for vaccine delivery owing to its advantages of high aqueous solubility and high charge density [162].

**Figure 7:**
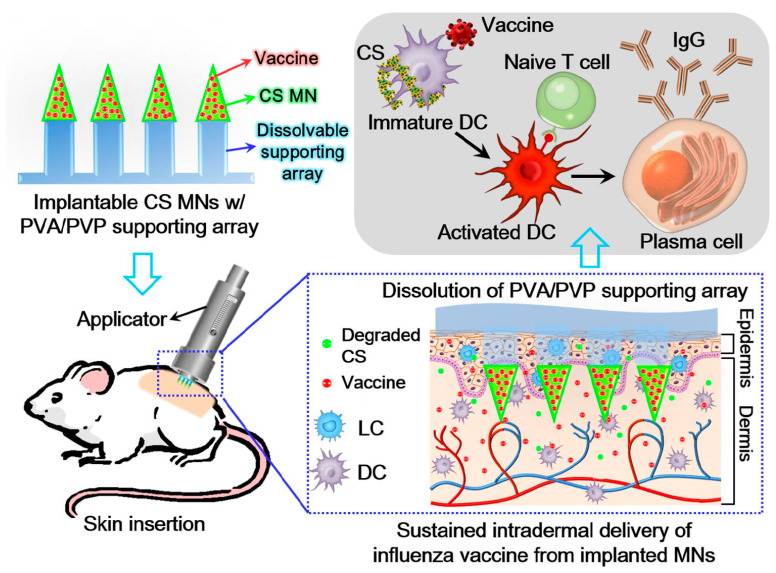
Schematic representation of the chitosan (CS) MNs for influenza vaccination delivery. The CS MN is easy to implant in the skin for sustained release of vaccines and immune activation [163]. Reproduced with permission from Yu-Hung Chen, Acta Biomaterialia; published by Elsevier, 2019.

CS MNs were used as intradermal delivery tools for vaccination (Figure 7). CS MNs have good mechanical strength, and the entire needle tip could reach the deep dermis. In addition to providing good mechanical strength, CS acts as an adjuvant to facilitate antigen uptake and presentation [163]. A composite MN with HA tip and CS base has also been used for long-lasting vaccine release. The fast release of antigen from the HA tip upon entry into the skin and the slow release of antigen from CS enhances the immunogenicity of the antigen. It produces a stable level of lgG antibodies for at least 16 weeks [164]. The dry-coated MN vaccine formulations reduce the demand for expensive cold-chain processes and facilitate the transmission of vaccines to rural areas [165].

### 5.2. Wound Healing

Wound healing is a sophisticated process, encompassing inflammatory, proliferative, and remodeling phases, involving good interactions between complex tissues and cells [166]. Polysaccharides exhibit unique advantages in promoting wound healing. In the early stages of inflammation, BSP stimulates the accumulation of inflammatory factors and exerts a healing effect on the wound. BSP could activate the expression of pro-inflammatory cytokines in M2 macrophages [167]. A BSP of 80 μg/mL induces human umbilical vascular endothelial cell proliferation and enhances VEGF and EGF expression [168]. CS could increase the level of anti-inflammatory factors (IL-10, TGF-β1) and decrease the level of pro-inflammatory factors [169]. Alginate could cause cytokine to arise produced by human monocytes, which facilitates tissue repair and promotes chronic wound healing [170].

**Figure 8:**
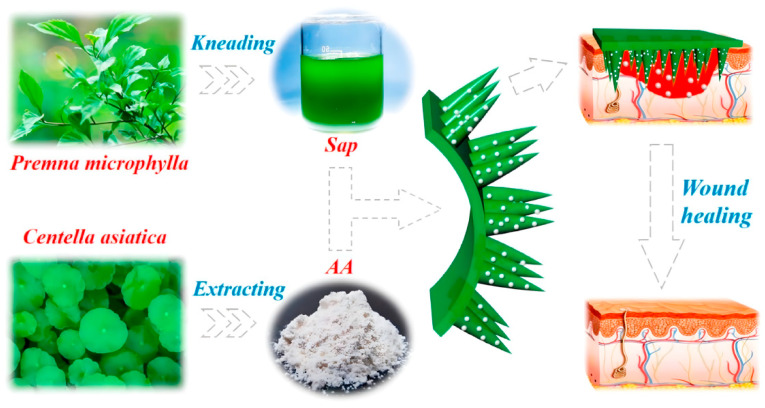
A schematic diagram exhibiting the application of Chinese herb MN (CHMN) patch made from *Premna microphylla* and *Centella Asiatica* extractions for wound healing [21]. Reproduced with permission from Junjie Chi, Bioactive Materials; published by KEAI, 2021.

Polysaccharides also act as delivery vehicles for active drugs to promote wound healing. Maltose MNs loaded with myrsinoside B exhibited good antioxidant and anti-inflammatory effects [171]. HA MNs loaded with green tea extraction (tea polyphenols) showed good antibacterial activity against Gram-positive and Gram-negative bacteria [172]. Pectin-rich *Premna microphylla* and Asiatic acid (AA), an extract of *CentellaaAsiatica*, have the abilities of anti-bacterial activity, and were utilized together to prepare the Chinese herb MN (CHMN) (Figure 8). The CHMN showed a better wound healing effect. Due to the good repair capacity of AA, the thickness of regenerated granulation tissue was up to 0.96 ± 0.12 mm, becoming the highest in the three experimental groups. CHMN significantly promoted the formation of new blood vessels and collagen in the wound [21].

### 5.3. Hypertrophic Scars

HSs are mainly due to the excessive collagen deposition of dermal fibroblasts, which often occur after wound healing. Shikonin, an active component extracted from Arnebiae Radix, was added to MN, which was made from soluble HA. Shikonin HA-MN was used to treat HSs. The results showed that Shikonin HA-MN had local therapeutic effects and was beneficial for local scar treatment in clinical practice. In addition, Shikonin HA-MN inhibited the expression of scar-related genes (TGF-β1, FAP-α, and COL1A1), providing a new approach to treating HSs [106]. Soluble HA MNs loaded bleomycin were also used to treat HSs [173]. Hydroxypropyl β-cyclodextrin (HP-β-CD) encapsulated with triamcinolone acetonide (TA) was co-loaded with verapamil (VRP) into carboxymethyl chitosan (CMCH), and BSP based MNs. The MN decreased the expression of the transforming growth factor-beta 1 (TGF-β1) and hydroxyproline (HYP) in HSs. The combination of TA and VRP showed a synergistic effect on the treatment of HSs [174].

**Figure 9:**
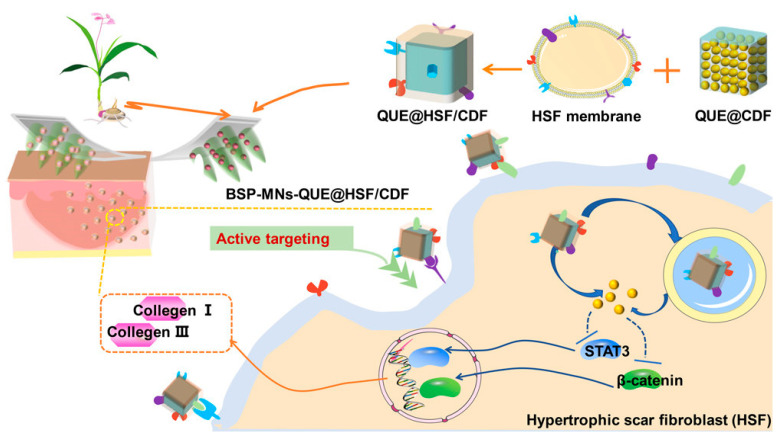
Schematic diagram of the administration of BSP-MNs-QUE@HSF/CDF, which can make active targeting hypertrophic scars (HSs). The HSs treatment efficacy was improved by reducing the expression of collagen I and III in HSs and regulating the pathways of β-catenin and STAT3 [98]. Reproduced with permission from Tong Wu, ACS nano; published by American Chemical Society, 2022.

The MN-mediated biomimetic transdermal system shown in Figure 9 was designed with a cyclodextrin metal-organic framework cross-linking with diphenyl carbonate (CDF). Quercetin (QUE) was loaded into the cyclodextrin metal-organic framework (CD-MOF) to prepare QUE-loaded CDF (QUE@CDF) and then coated with an HSF membrane (QUE@HSF/CDF). Then, QUE@HSF/CDF was dispersed in BSP-based MN to achieve targeted delivery. BSP showed synergistic effects, and the mechanical strength was superior to HA-based MN. This system reduced the expression of collagens I and III in HSs to improve the treatment efficacy of HSs, and MNs prepared in this way had better mechanical strength than HA-based MNs [98].

### 5.4. Psoriasis

**Figure 10:**
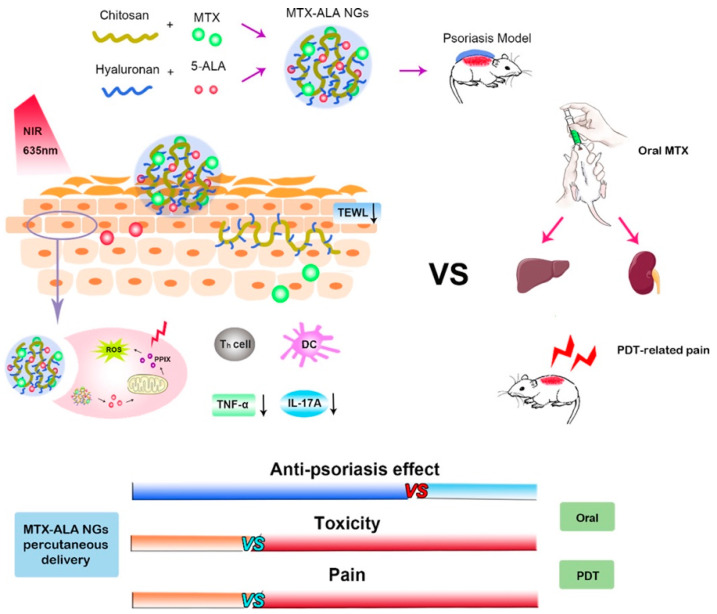
Schematic diagram of combined chemical-photodynamic treatment of psoriasis with MTX and ALA loaded CS/HA nanogels (NGs). MTX-ALA NGs promoted the penetration of both ALA and MTX into the skin, synergistically enhancing the local treatment of psoriasis and reducing the toxicity and pain associated with the photodynamic therapy of ALA and oral MTX [177]. Reproduced with permission from Yixuan Wang, Carbohydrate Polymers; published by Elsevier, 2022.

Psoriasis is a chronic degenerative inflammatory disease with multiple signs caused by a mixture of genetic and environmental factors [175]. There are many therapies available for psoriasis. Methotrexate (MTX) is the most commonly adopted drug for psoriasis treatment. Side effects such as stomatitis and gastrointestinal discomfort may occur when MTX is administered orally or through the parenteral route. The high molecular weight and hydrophilic nature of MTX make it less effective in passively diffusing through the stratum corneum. To overcome this problem, the MTX-HA MN was prepared. The good hydration ability of HA allows MTX to stay in the epidermis and reduces penetration into the deeper skin [176]. The overexpression of CD44 protein in psoriatic skin is used as a potential target to treat psoriasis. CS/HA nanogels loaded with MTX and ALA (MTX-ALA NGs) exhibited good synergistic therapeutic effects (Figure 10).

On the one hand, CS and HA enabled the nanogels to have cellular uptake enhancement and targeting ability for psoriasis. On the other hand, the nanogels exhibited characteristics shared by nanoparticles and hydrogels, enhanced drug penetration, and high loading capacity. MTX-ALA NGs effectively downregulated the pro-inflammatory cytokines of IL-17A and TNF-α, reduced the side effects of oral MXT, and enhanced MXT and ALA penetration and deposition in the skin [177]. CS nanoparticles loaded with tacrolimus utilized the positive charge of CS to combine with the negative charge sites of the skin, enhancing the deposition rate of tacrolimus in the skin, with 82.0% ± 0.6 of the drugs retained in the skin. Its therapeutic efficacy is superior to commercially available tacrolimus^®^ ointment [157].

### 5.5. Skin dendritic Cell Activation

**Figure 11:**
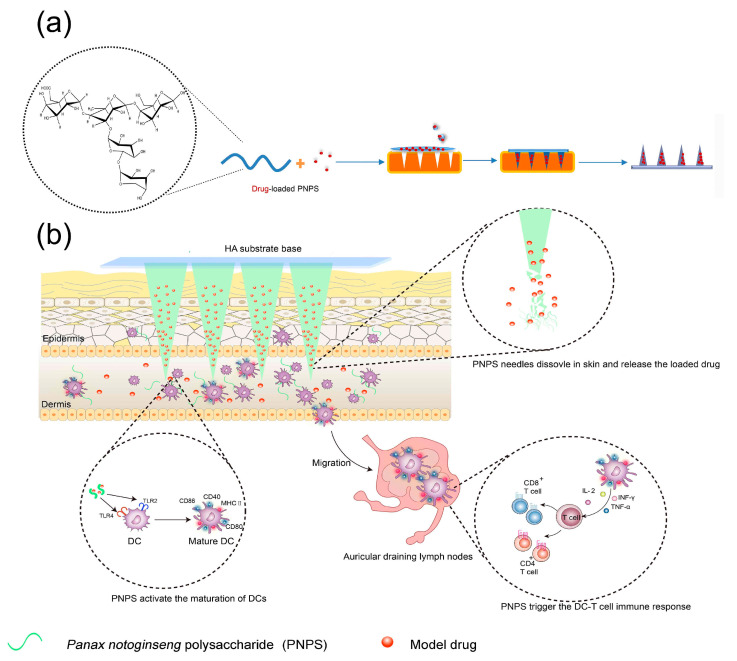
Schematic diagram of the preparation of the dissolved *Panax notoginseng* polysaccharides MN (PNPS MNs) and in vivo activation process. (**a**) The preparation process of PNPS MNs loaded with the model drugs. (**b**) PNPS MNs dissolved and activated skin dendritic cells and triggered DC-initiated T cell immune response for transcutaneous immunization [99]. Reproduced with permission from Chengxiao Wang, Carbohydrate Polymers; published by Elsevier, 2021.

Immune effector DCs are important antigen-presenting cells (APC) that are associated with adaptive and innate immunity and target cancer immunotherapy and vaccine adjuvants [178]. Mature DCs are the only cells that can directly communicate with T cells and trigger their proliferation to generate cellular immunity. The skin contains quantities of epidermal DCs, which recognize and deliver antigens to lymph nodes, thereby triggering a response. Skin DCs activation is of great immunological importance. PNPS, isolated from the traditional Chinese herb *Panax notoginseng*, significantly induces the maturation of bone-marrow-derived DCs (BMDCs). The PNPS MN was prepared for delivery of PNPS (Figure 11). PNPS MNs could easily cross the stratum corneum and diffuse to the depth of 450 µm, allowing good targeting of skin DCs. In addition, PNPS with biological activity could identify and target skin DC through Toll-like receptor 2 (TLR2)/Toll-like receptor 4 (TLR4) and trigger the maturation of DC. Administration via PNPS MNs demonstrated a higher ratio of CD11c+/FTSC+ DCs cells, 7 and 2.5 times that of PNPS solutions and dextran MNs [99].

### 5.6. Insulin

Insulin is the most powerful drug for regulating the level of blood glucose in patients with type I diabetes. However, the transdermal route has become the favored insulin administration due to the low absorption or enzymatic degradation of insulin in the liver. Usually, insulin is administered through a transdermal needle, but this is painful and inconvenient, often causing poor patient compliance [179]. Therefore, polysaccharide-based MN systems have been used to deliver insulin, such as HA [180], alginate [181], and maltose [56]. Insulin MN patches prepared from gelatin and starch have sufficient mechanical strength and dissolve completely after five minutes of insertion into the skin [179]. Pullulan polysaccharide (PL), a non-ionic natural occurring exopolysaccharide produced by yeasts, was adopted to prepare PL microneedle (PLMN) for insulin delivery. It can be stored at 4, 20, and 40 °C for at least one month to ensure insulin activity, which enables insulin to be preserved for a long time and is of great significance for the use of insulin in remote areas [182].

**Figure 12:**
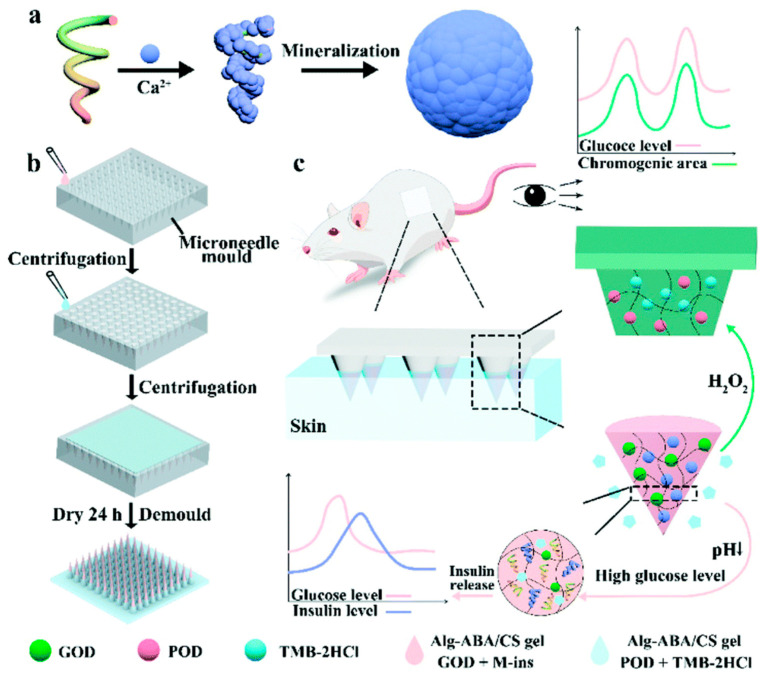
Schematic diagram of glucose responsiveness that triggers the self-adjustable release of insulin and perception of blood glucose in real-time. (**a**) Insulin mineralization under Ca^2+^. (**b**) Dual-functional MN prepared by cross-linking and micro-molding. (**c**) Self-regulated release of insulin is triggered by glucose responsiveness and real-time glucose sensing [108]. Reproduced with permission from Xuetong Sun, Biomaterials Science; published by The Royal Society of Chemistry, 2022.

In addition to enhancing insulin preservation time in vitro, smart insulin MN patches are also being developed. An MN patch that allowed visualization and quantification of blood glucose and self-regulation of insulin release was investigated (Figure 12). MNs were prepared by cross-linking chondroitin sulfate and 3-aminophenyl boronic acid (ABA)-modified sodium alginate, loaded with mineralized glucose oxidized (GOD) and insulin particles. H^+^ produced by the catalytic reaction of GOD with glucose progressively dissolved mineralized insulin particles, leading to the self-regulated release of insulin. The increasing level of H_2_O_2_ resulted in a visible color change, which allowed for a reading of the glucose content changes [108]. The dynamically capped hierarchically porous MNs, which utilized the dissolution of CS hydrogels, allowed for the intelligent release of insulin [102]. Smart MN patches offer a new perspective in the self-adjustable insulin release field. To prevent hypoglycemia caused by the overuse of insulin, methacrylate hyaluronic acid (MeHA)-based smart insulin MNs were used to automatically deliver glucagon at low glucose concentrations. This smart MN patch shifts the treatment of hypoglycemia from emergency treatment to a preventive measure, enhancing patient safety [183].

### 5.7. Immunotherapy

**Figure 13:**
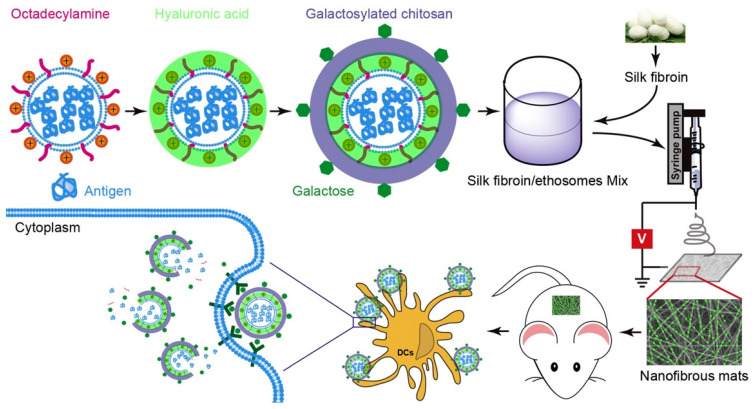
A schematic diagram of the preparation of the Eth-HA-GC/SF loaded OVA, causing cellular and humoral immune responses in the mouse model [187]. Reproduced with permission from Xingxing Yang, Journal of Controlled Release; published by Elsevier, 2020.

Transdermal immunotherapy exhibits better results than oral administration and injection due to a large number of APCs in the skin. Polysaccharides have certain specific target cells that show unique advantages in immunotherapy. The main mechanism by which polysaccharides perform immunomodulation is usually considered to be through activation of the body’s immune response [184]. Polysaccharides could activate immune cells such as T lymphocytes and macrophages to exert immune activity [185]. HA with galactosylated chitosan (GC) modified ES (Eth-HA-GC) was loaded on silk fibroin (SF) nanofiber mats (Eth HA-GC/SF) to perform transdermal immunization (Figure 13). Galactosyl is thought to be able to target DCs [186]. Eth-HA-GC/SF can target and induce DCs maturation. Eth-HA-GC/SF loaded with OVA can increase the expression of marker molecules (CD80, CD86) associated with DCs maturation in BMDCs and improve the expression of IFN-γ and IL-6 in spleen cells. Eth-HA-GC/SF is considered to have the good immunotherapeutic potential [187]. Chemically modified polysaccharides often exhibit immunomodulatory capabilities. Sulfated polysaccharides can promote interleukin secretion by macrophages [188], acetylated polysaccharides can enhance antioxidant properties [189], and carboxymethylated polysaccharides can enhance the ability to induce maturation of DCs [190]. In general, polysaccharides show promising potential in immunotherapy.

### 5.8. Skin Cancer

**Figure 14:**
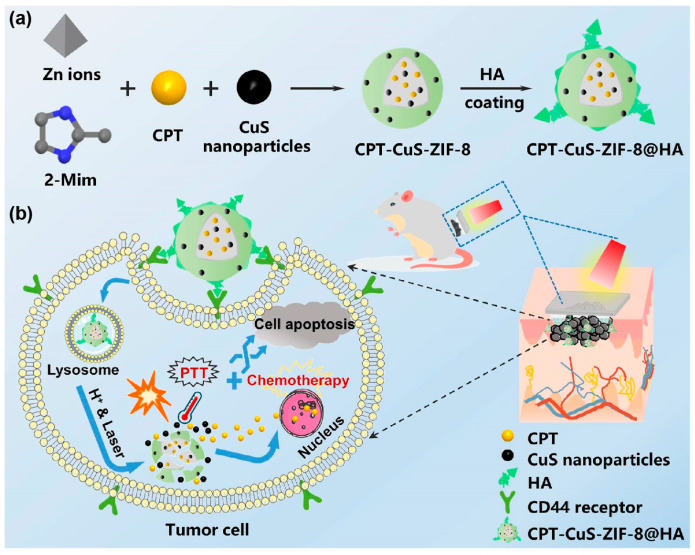
Schematic representation of (**a**) the preparation of CPT-CuS-ZIF-8@HA and (**b**) multifunctional nanoparticle-integrated DMNs for transdermal drug delivery, smart drug release, targeted delivery, and synergistic chemotherapy-photothermal therapy in melanoma [105]. Reproduced with permission from Yiting Zhao, Acta Biomaterialia; published by Elsevier, 2021.

Malignant melanoma is a fatal type of skin cancer. To reduce the side effects caused by the systemic application of anticancer drugs, polysaccharide-based transdermal drug delivery systems provide a new tactic for the effective treatment of skin cancer. Astragalus polysaccharide was shown to treat melanoma by inducing programmed death-ligand 1 (PD-L1) downregulation [191]. DOX, an anthracycline drug, has been successfully used to treat several cancers. Carboxymethylcellulose (CMC), a cellulose polysaccharide derivative, formed nanocomplexes with DOX. The electrostatic interactions stabilized the anionic carboxylate group of CMCs and the cationic amino group of DOX. The degree of substitution of CMC was shown to influence the DOX release. The CMC-DOX nanocomplexes with citric acid hydrogels could control the drug release [192]. A multifunctional nanoparticle-integrated soluble MN, called CPT-CuS-ZIF-8@HA@MN, was prepared for the treatment of malignant melanoma (Figure 14). Nanoparticles were prepared by adding a photothermal agent (CuS) to Zeolitic imidazolate framework-8 functionalized (ZIF-8) by HA. ZIF-8, a promising drug carrier for tumor therapy, could be modified to enhance active targeting capability [193]. HA itself could target CD44, and ZIF-8, modified by HA, could specifically gift down cellular uptake to enhance therapeutic efficacy. The MNs could be loaded with multiple drugs simultaneously, improving the specificity of targeting tumors and overcoming the limitation of monotherapy [105].

### 5.9. Rheumatoid Arthritis

Rheumatoid arthritis (RA) is a chronic inflammatory disease that affects the joints. Polysaccharides and polysaccharides-based nanoparticles have been widely researched in RA treatment [194]. Dendrobium huoshanense stem polysaccharide treats RA by inhibiting inflammatory signaling pathways [195]. Berberine encapsulated in CS, a surface-modified bilosome nanogel (BER-CTS-BLS), was used for the treatment of RA. BER-CTS-BLS has a size of 202.3 nm and has high drug encapsulation and good stability. The positive charge and bioadhesive properties of BER-CTS-BLS allowed the permeability coefficient of BER-CTS-BLS to be 1.5 times higher than that of BER solution, achieving better drug diffusion. BER-CTS-BLS gel significantly decreased the swelling percentage of rat paw edema after 12 h, providing a new therapeutic approach for the treatment of RA [196]. Cationic starch and poly (vinyl alcohol) (PVA)-based films loaded with MTX were used for the treatment of RA, avoiding the intestinal side effects caused by MTX. The films demonstrated good drug distribution and drug loading ability (>68.4%) [197].

**Figure 15:**
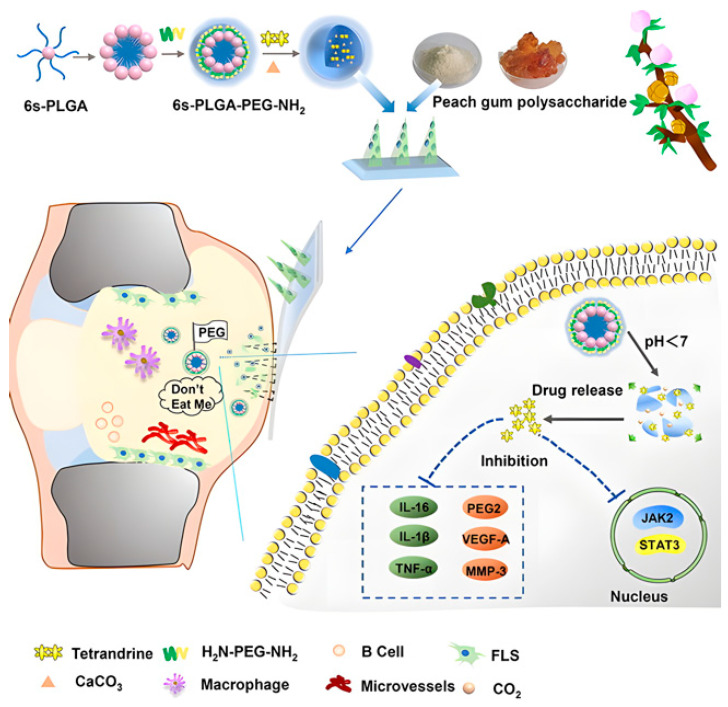
Schematic representation of the preparation of acid-responsive released [6s-NPs (CaCO_3_)] MN and the in vivo regulation of the VEGF, JAK2, and STAT3 pathways for the treatment of Rheumatoid Arthritis (RA) [198]. Reproduced with permission from Hu, Hongmei, Chemical Engineering Journal; published by Elsevier, 2022.

Acid-responsive nanoparticles could enhance the transdermal treatment of RA. PEGylated star-shaped PLGA, hybridized by the calcium carbonate, formed nanoparticles [6 s-NPs (CaCO_3_)], which increase the loading of tetrandrine (Tet). Peach gum polysaccharides (GPs) secreted from peach trees exhibit good antioxidant and antibacterial activities. The [6s-NPs (CaCO_3_)] were loaded into MN prepared from GPs (GP-MN). GP-MN exhibited good transdermal effects and better mechanical strength than HA-MN (Figure 15). This delivery method increases Tet’s synovial uptake and improves the regulation of the VEGF, JAK2/p-JAK2, and STAT3/p-STAT3 pathways [198].

### 5.10. Others

In addition to the aforementioned disease treatment, polysaccharides are extensively employed in treating various illnesses for their distinctive advantages. Pectin-based silver nanocomposite films loaded with donepezil were used to treat Alzheimer’s disease, where nanosilver and pectin were compounded to improve the absorption and release of the drug, exhibiting good antibacterial properties [199]. Sodium carboxymethylcellulose (SCMC)-based MNs loaded with calcitonin gene-related peptide (CGRP), a neuropeptide released from sensory nerve terminals, were used as a safe and convenient way to treat neuropathic pain [200]. Trimethyl CS/sodium alginate multilayer nanomembranes encapsulating pentoxifylline (PTX) were used as a new modality to treat chronic venous ulceration [201]. In terms of disease treatment, polysaccharide-based transdermal drug delivery demonstrates its unique advantages and provides a new approach to disease treatment.

## 6. Conclusions

Polysaccharides, as natural polymers, have been extensively employed in transdermal drug delivery systems. Polysaccharides derived from herbal, marine, and microbial sources show unique advantages, such as antibacterial, biodegradable, anti-inflammatory, antioxidant, and non-toxic. On top of reducing gastrointestinal side effects, avoiding hepatic first-pass metabolism, and improving patient compliance, polysaccharide-based transdermal drug delivery systems show improved drug targeting, safety, and biocompatibility.

Polysaccharide-based vehicles also demonstrate better properties than traditional polymers, including (1) better hydrophilicity and swelling properties, stimulus-responsive hydrogel shows better therapeutic results; (2) better tensile strength, and polysaccharide-based composite films exhibit better biocompatibility and drug synergy effects; (3) enhanced mechanical strength and controlled drugs release by cross-linking and modification of polysaccharides-based MNs; (4) reduce the hindrance of the “brick and mortar” structure of the stratum corneum by hydration and charge effects and therefore improve the drug penetration efficiency. In addition, polysaccharide-based nanoparticles have shown advantages in the treatment of diseases, including (1) natural targeting ability. They can be used to target specific receptors and deliver drugs to the treatment site ((2) improve penetration ability). They act as a carrier to help deliver drugs to the site of action, improving the utilization of the drug ((3) natural pharmaceutical activity). Some polysaccharides exhibit natural an-bacterial and anti-inflammatory ability in the treatment of skin diseases; ((4) better patient compliance). Polysaccharides are biodegradable, which greatly improves patient compliance.

It Is foreseeable that polysaccharides-based transdermal drug delivery systems will become a promising way to deliver drugs. They are combining with nanotechnology to prepare “smart” formulations. In the future, as an alternative to the oral route, improving portability and acceptability for patients will be essential for further development.

## Data Availability

Not applicable.
